# Bilateral Thalamic Stroke: A Case of COVID-19 Vaccine-Induced Immune Thrombotic Thrombocytopenia (VITT) or a Coincidence Due to Underlying Risk Factors?

**DOI:** 10.7759/cureus.18977

**Published:** 2021-10-22

**Authors:** Richard Giovane, Jessica Campbell

**Affiliations:** 1 Family Medicine, University of Alabama (UAB), Greenville, USA; 2 Family Medicine, Jackson Hospital, Greenville, USA

**Keywords:** vaccine preventable diseases, heparin induced thrombocytopenia (hit), vaccine-induced thrombotic thrombocytopenia (vitt), covid 19, bilateral thalamic disease

## Abstract

Vaccine-induced immune thrombotic thrombocytopenia (VITT) is a rare but potentially life-threatening side effect that has only been observed in adenovirus-based vaccines for coronavirus disease 2019 (COVID-19). VITT is an immune-mediated condition that generally presents within five to 10 days post-vaccination with thrombosis, thrombocytopenia, and coagulation abnormalities. A diagnosis of VITT is made clinically and through laboratory testing. Although VITT is an important differential to consider, it is believed that more emphasis should be placed on vaccination due to the safety and efficacy in overcoming COVID-19.

## Introduction

Severe acute respiratory syndrome coronavirus 2 (SARS-CoV-2) is a coronavirus that was first identified in Wuhan in December 2019. It caused an outbreak of pneumonia, which was then classified as coronavirus disease 2019 or COVID-19. It subsequently spread to the rest of the world, causing a global pandemic. As of September 2021, the WHO estimates a total of 228 million cases of COVID-19 with 4.6 million deaths worldwide [1]. As clinical trials are still ongoing regarding the pharmacological treatment of COVID-19; vaccination is currently one of the only proven preventative measures against COVID-19, as the efficacy of the vaccine ranges from 70-95%, depending on the vaccine type [2]. The safety of administering this vaccine has been very rigorous and is under constant surveillance through the Vaccine Adverse Event Reporting System (VAERS) [3]. Despite the rigorous testing and measures of safety, there have been reported side effects of the vaccine from mild to severe. Mild side effects include fever, chills, myalgia, and headaches; however, these side effects are transient and do not pose a risk to the patient [4]. mRNA platform vaccines, such as the Pfizer and Moderna vaccines, have had case reports of myocarditis and anaphylaxis but these instances have been rare [5-6]. The adenovirus recombinant vaccine from AstraZeneca and Janssen also has a known but very extremely small risk of developing vaccine-induced immune thrombotic thrombocytopenia (VITT), which is a prothrombotic syndrome that resembles heparin-induced thrombocytopenia (HIT) [7]. Currently, there have not been any defined risk factors for VITT [7]. Although rare, VITT imposes an increase in mortality if not recognized and treated promptly. Such rare side effects have caused public concern and hesitancy in getting vaccinated, which slows down our ability to overcome COVID-19 as well as prevent further variants from spreading.

## Case presentation

**Figure 1:**
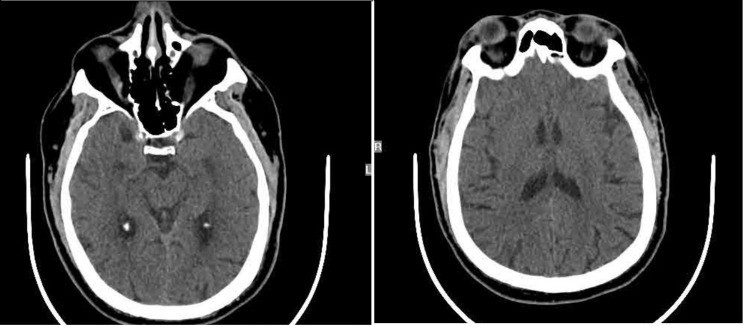
CT scan of the head without contrast

**Figure 2:**
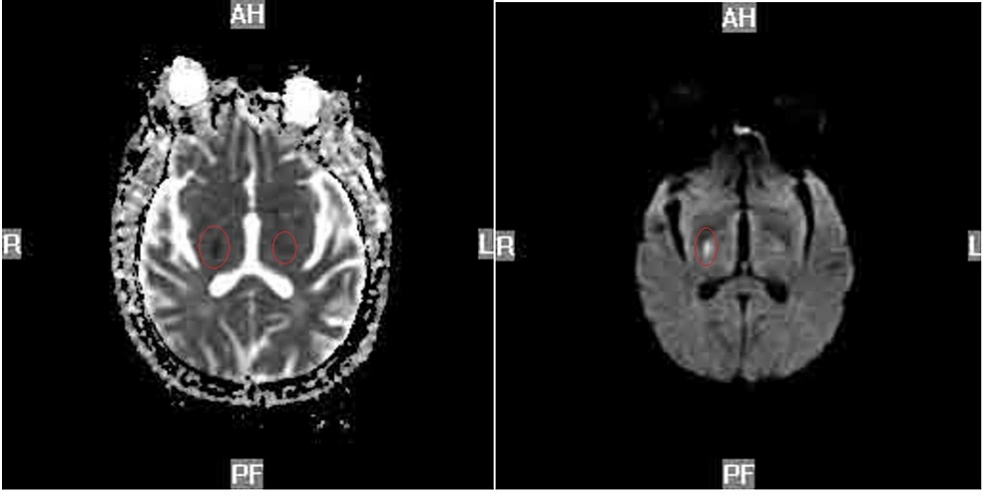
Diffusion-weighted MRI head (left). Axial MRI of the head (right) Note: Poor quality due to patient's hemiballismus

We report a case of a 62-year-old male with a past medical history of hypertension, hyperlipidemia, type 2 diabetes, low back pain, and end-stage renal disease for seven years, with dialysis on Mondays, Wednesdays, and Fridays, who presented to the emergency room with complaints of left-sided weakness in his arm and leg for one day. The patient reports that the day before, he woke up without any issues and went to his local drug store to get his first dose of Pfizer COVID-19 vaccine. He received the vaccine in his right arm and then visited his pain management doctor for a follow-up of his lower back pain. The patient then went home and visited a friend. He reported that around 4 pm while talking with his friend, his left arm and leg felt “weak” and his left leg “gave out” on him. When asked further, the patient reported that his leg “just wouldn’t move and felt numb;” however, regarding his left arm, the patient reported that it “just felt numb” but he could move it. The patient did not seek medical attention at this time, as he reported his friend drove him back home and his symptoms completely resolved by 9 pm. The patient then went to dialysis the next day. During dialysis, he reported that his left arm and leg went completely numb. He then reported slurred speech and he “couldn’t control his left leg.” When clarifying this with the patient, his leg was not making any purposeful movement. His vitals were taken at the time of the event, which showed a blood pressure of 180/90, heart rate (HR) of 92, respiratory rate (RR) 16, and temperature of 98.7F. The patient was sent to the emergency room for further workup. While in the emergency room, the patient was worked up for a transient ischaemic attack (TIA) versus stroke, as he still had slurred speech, left facial droop, and hemiballismus in his left lower extremity. His initial head CT without contrast did not show any ischemic or hemorrhagic stroke (Figure 1). A chest X-ray was done, which showed a large cardiac silhouette; however, there were no other acute findings. A tele-neurology consult was also done and per neurology recommendations, the patient did not meet the criteria for tissue plasminogen activator (tPA), and it was suggested that the patient have an MRI. Since the patient did have hemiballismus, an MRI without contrast was challenging and therefore limited to diffusion-weighted and apparent diffusion coefficient (ADC) sequencing (Figure 2). MRI showed bilateral acute ischemic changes involving both thalami with right larger than left. The patient had no prior surgeries and denied alcohol, drug, or tobacco use. He did have a family history of diabetes mellitus type 2 and hypertension. He did not have a family history of bleeding disorders.

On physical exam, the patient had a Glasgow coma scale of 13. His cardiac exam revealed an S1S2 without any murmur, lungs were clear bilaterally, and the abdominal exam was unremarkable.

For the patient’s neurological exam, he did have a left facial droop with dysarthria; however, he did not have anomic aphasia. The patient’s cranial nerves were tested as well (Table 1). For cranial nerves (CN) 2, 3, 4, and 6, a penlight was used, and it showed anisocoria with the left being bigger than the right, but they were both reactive to light. Also, he had full EOM but horizontal nystagmus was noted bilaterally. CN5 was preserved, as he was able to feel a wisp of cotton in all three regions in his face bilaterally. CN7 was tested by asking the patient to smile, wrinkle his forehead, puff out both of his cheeks, and close his eyes tightly. This exam showed weakness in his left side with a left facial droop, but he was able to wrinkle his forehead on his left side; the right side was unremarkable. CN 8, 9, 10, and 11 were tested via a hearing test, uvula examination, and telling the patient to shrug his shoulder,s respectively. All of these were preserved. CN 12 was tested by asking the patient to stick out his tongue. It showed that the tongue slightly deviated to the right side. His right arm and leg strength was 5/5, with preserved sensation. His deep tendon reflexes (DTR) on the right side were 2+ in his biceps, triceps, patella, and Achilles tendons. Left-sided arm strength was 1/5 with preservation of sensation and DTR was 2+. His left leg did have hemiballismus with preserved sensation and DTR was unable to be tested.

**Table 1:** Neurological exam in the emergency room CN: cranial nerve

Neurological Exam	Findings
CN II, III, IV, VI - Visual acuity and ocular movements	-Anisocoria with the left being bigger than the right. -Horizontal nystagmus bilaterally.
CN V - Light touch	-Preserved bilaterally
CN VII- Facial muscles	-Left facial droop, able to wrinkle his forehead -Right side preserved
CN VIII - Whisper test	-Preserved bilaterally
CN IX, X - Uvula examination	-Preserved
CN XI - Shoulder strength	-Preserved bilaterally
CN XII - Tongue movement	- Tongue slightly deviated to the right
UE/LE strength and sensation	-Right and leg strength were 5/5, with preserved sensation and DTR 2+. - Left-sided arm strength was 1/5 with preservation of sensation and DTR 2+. -Left leg noted to have hemiballismus with preserved sensation

The patient had blood work done as well (Table 2). A complete blood count (CBC) was done, which showed a low platelet count of 116 10^3uL with the rest of the CBC unremarkable. A comprehensive metabolic panel (CMP) showed an elevated blood urea nitrogen (BUN) of 42 mg/dL and creatinine of 7.9 mg/dL with other labs unremarkable. The elevated BUN and Cr were at baseline due to his end-stage renal disease (ESRD). His a1c was 6.5 and a lipid panel showed a cholesterol of 292 mg/dL, triglycerides of 198 mg/dL, and a very-low-density lipoprotein (VLDL) of 39 mg/dL. His prothrombin time (PT)/international normalized ratio (INR) and activated partial thromboplastin time (aPTT) were within normal limits. His COVID-19 test was negative, and he was never infected with COVID-19 previously.

**Table 2:** Blood work in the emergency room MCV: mean corpuscular volume; BUN: blood urea nitrogen; GFR: glomerular filtration rate; ALT: alanine transaminase; AST: aspartate aminotransferase; HDL: high-density lipoprotein; LDL: low-density lipoprotein; VLDL: very low-density lipoprotein

WBC	6.6 10^3/uL
RBC	4.02 10^6/uL
Hemoglobin	11.8 g/dL
Hematocrit	35.6%L
MCV	89 fL
Platelets	116 10^3/uL
Glucose	111 mg/dL
BUN	42 mg/dL
Creatinine	7.90 mg/dL
Sodium	138 mmol/L
Potassium	4.4 mmol/L
Chloride	100 mmol/L
CO2	27 mmol/L
Calcium	8.2 mg/dL
GFR	9 ml/min
ALT	16 IU/L
AST	23 IU/L
A1C	6.5
Fibrinogen	458
D-dimer	150 ng/ml
Cholesterol	292 mg/dL
Triglycerides	198 mg/dL
HDL	35 mg/dL
LDL	217 mg/dL
VLDL	39 mg/dL

The patient was admitted for close observation in the ICU and further workup. He was placed on clopidogrel 75 mg and aspirin 81 mg and started on lovenox for DVT prophylaxis. Heparin was avoided due to the patient’s presentation and differential of possible VITT. While in the ICU, the patient had a carotid ultrasound, which showed no stenosis, and an echocardiogram showed an ejection fraction (EF) of 60% with mild left ventricular hypertrophy but otherwise no anatomical issues. Permissive hypertension was allowed, and he was on sliding-scale insulin for tight blood glucose regulation. A D-dimer was ordered as well as platelet factor 4 (PF4) and fibrinogen level to rule out VITT. Nephrology was also consulted to initiate his dialysis. His blood pressure remained elevated after 24 hrs, and he was restarted on his daily hydrochlorothiazide 50 mg and Norvasc 10 mg, and nephrology added losartan 25 mg, which stabilized his blood pressure. The patient was hospitalized for a total of five days. His hemiballismus resolved by hospital Day 3, but his left upper and lower extremity strength did not improve. His speech slightly improved and was more comprehensible. Regarding his blood work, his platelet count did improve to 134 10^3uL on Day 2 then remained at 154 10^3uL. His D-dimer was 150 ng/ml, fibrinogen was 458 mg/dL, and PF4 activity was negative per enzyme-linked immunoassay (ELISA). The patient was eventually discharged to 21-day rehab where his left lower extremity strength improved to 3/5; however, left upper extremity strength remained 1/5. The patient followed up one week later with his primary care provider in which it was noted that his speech improved; however, strength in both extremities remained the same.

## Discussion

VITT is an immune-mediated condition in which immunoglobin G antibodies are formed against PF4 [7-8]. These IgG antibodies activate platelets by binding to FcγIIa, which induces a pro-coagulative state [8]. Although similar to HIT, VITT occurs in the absence of heparin. VITT has only been reported in the adenovirus recombinant vaccines AstraZeneca and Janssen [9]. The incidence is very rare, with an estimated 1 in 533,333 in the United States [10]. A few risk factors for developing VITT have been reported such as being female and being less than 60 years old; however, with such few cases, it is difficult to determine if these are true risk factors due to the limited sample size [7,11].

Patients with VITT generally present within five to 10 days post-vaccination with thrombosis, thrombocytopenia, and coagulation abnormalities [7-8]. Thrombosis is the most common feature, is either venous such as cerebral venous thrombosis (CVT), splanchnic, adrenal, and PE, or it can be arterial and cause ischemic stroke in unusual locations, limb ischemia, or myocardial infarction [9,11]. Thrombocytopenia is another abnormality found on presentation as well as an elevated D-dimer and decreased fibrinogen [7-9].

The diagnosis of VITT is made through laboratory testing and clinically. Patients will have underlying thrombocytopenia, an elevated D-dimer, and low or normal fibrinogen levels. Patients must also have had the vaccine within five to 10 days although symptoms have been known to develop before Day 5. A positive PF4 antibody on ELISA confirms the diagnosis; however, in most cases, this is not available in hospitals so if VITT is suspected, it must be treated as soon as possible. Although treatment options are emerging, treatment is aimed at anticoagulating the patient to prevent further thrombus formation. Initially, non-heparin anticoagulants were preferred; however, recent studies have shown no difference in outcomes with heparin anticoagulants versus non-heparin anticoagulants [8,11]. If there is a high degree of suspicion of VITT in the setting of thrombocytopenia and thrombosis or if the patient just has thrombosis, the patient is anticoagulated and is treated empirically with intravenous immunoglobulin (IVIG) 1 g/kg daily for two days [11-12]. Platelet transfusion is also avoided and only reserved for bleeding. If there is only thrombocytopenia, treatment involves anticoagulation and escalating treatment with IVIG once there is a confirmed positive ELISA test. Plasma exchange has also been proposed for patients with VITT with refractory treatment. Plasma exchange is effective, as it removes anti-PF4 antibodies and excessive inflammatory cytokines [13]. Patients can be discharged once platelets are >50,000 10^3 uL, have no bleeding, and are stable on anticoagulants [11].

In our case, the patient did present with a bilateral thalamic stroke, which is rare, as it accounts for only 0.6% of all first-ever ischemic strokes [14-15]. Risk factors for thalamic strokes include hypertension, type 2 diabetes, ESRD, and hyperlipidemia, all of which the patient had [15]. Fortunately, the initial work-up for VITT in our patient was negative and confirmatory PF4 ELISA was negative, however, there was a degree of suspicion for VITT on initial presentation due to the cerebrovascular accident (CVA) being bilateral and the timeline in which symptoms occurred in accordance with his COVID-19 vaccine. Given this, however, the patient had several risk factors that led to his stroke, and it is believed that the patient receiving the COVID-19 vaccine and then developing symptoms was coincidental. As there has been public concern regarding the COVID-19 vaccine and the potentially rare side effects, clinicians should strongly encourage everyone who is able to do so to get vaccinated, as the benefits heavily outweigh the minuscule risks.

## Conclusions

This case is important, as there has been a recent hesitancy in the public toward vaccination due to an over-emphasis within the media of extremely rare side effects from the COVID-19 vaccine. VITT is a rare, life-threatening condition that occurs within five to 10 days post-vaccination. It presents with thrombosis, thrombocytopenia, and coagulation abnormalities and should be ruled out in patients presenting with these symptoms in the setting of recent COVID-19 vaccination. Although the incidence is low and has only been observed in adenovirus-based vaccines, a systemic review of VITT is encouraged, when enough cases are available in the future, to identify risk factors, morbidity, and the occurrence of VITT in other COVID-19 vaccine types. Regardless of the low risk of VITT, clinicians should emphasize encouragement toward vaccination in all individuals who are able to do so.
